# Genetic and Physiological Characterization of Fructose-1,6-Bisphosphate Aldolase and Glyceraldehyde-3-Phosphate Dehydrogenase in the Crabtree-Negative Yeast *Kluyveromyces lactis*

**DOI:** 10.3390/ijms23020772

**Published:** 2022-01-11

**Authors:** Rosaura Rodicio, Hans-Peter Schmitz, Jürgen J. Heinisch

**Affiliations:** 1 Departamento de Bioquímica y Biología Molecular, Instituto Universitario de Biotecnología de Asturias, Universidad de Oviedo, 33006 Oviedo, Spain; mrosaura@uniovi.es; 2 Fachbereich Biologie/Chemie, Universität Osnabrück, AG Genetik, Barbarastrasse 11, D-49076 Osnabrück, Germany; hans-peter.schmitz@uni-osnabrueck.de

**Keywords:** glycolysis, pentose phosphate pathway, oxidative stress, moonlighting enzymes

## Abstract

The milk yeast *Kluyveromyces lactis* degrades glucose through glycolysis and the pentose phosphate pathway and follows a mainly respiratory metabolism. Here, we investigated the role of two reactions which are required for the final steps of glucose degradation from both pathways, as well as for gluconeogenesis, namely fructose-1,6-bisphosphate aldolase (FBA) and glyceraldehyde-3-phosphate dehydrogenase (GAPDH). In silico analyses identified one gene encoding the former (*KlFBA1*), and three genes encoding isoforms of the latter (*KlTDH1*, *KlTDH2*, *KlGDP1*). Phenotypic analyses were performed by deleting the genes from the haploid *K. lactis* genome. While *Klfba1* deletions lacked detectable FBA activity, they still grew poorly on glucose. To investigate the in vivo importance of the GAPDH isoforms, different mutant combinations were analyzed for their growth behavior and enzymatic activity. KlTdh2 represented the major glycolytic GAPDH isoform, as its lack caused a slower growth on glucose. Cells lacking both KlTdh1 and KlTdh2 failed to grow on glucose but were still able to use ethanol as sole carbon sources, indicating that KlGdp1 is sufficient to promote gluconeogenesis. Life-cell fluorescence microscopy revealed that KlTdh2 accumulated in the nucleus upon exposure to oxidative stress, suggesting a moonlighting function of this isoform in the regulation of gene expression. Heterologous complementation of the *Klfba1* deletion by the human *ALDOA* gene renders *K. lactis* a promising host for heterologous expression of human disease alleles and/or a screening system for specific drugs.

## 1. Introduction

Glycolysis has been studied for more than a century, with a crucial impact on biochemistry, ever since the observation of cell-free fermentation in extracts from the wine, beer, and baker’s yeast *Saccharomyces cerevisiae* [1]. For this yeast, glycolysis is essential, as a lack of enzyme activity in most steps leads to growth inhibition on glucose as a sole carbon source [2,3,4]; reviewed in [5]. *S. cerevisiae* is highly specialized in alcoholic fermentation, to which it owes its biotechnological importance. To this end, the reactions of pyruvate decarboxylase and alcohol dehydrogenase convert the glycolytic end-product pyruvate into carbon dioxide and ethanol [6,7].

In humans, glycolysis is also of utmost importance in central carbohydrate metabolism, as exemplified by so-called glycogen-storage diseases associated with impaired enzyme activities, e.g., phosphofructokinase (glycogen-storage disease VII; Tarui’s disease [8]) or fructose-1,6-bisphosphate aldolase (glycogen-storage disease XII [9]). Moreover, cancer cells generally increase the flux through glycolysis, designated as the so-called Warburg effect, which has been compared to the metabolism of *S. cerevisiae*, a Crabtree-positive yeast that ferments sugars even if oxygen is available [10,11]. While *S. cerevisiae* may thus serve as a model organism to study the physiology of human cancer cells, it is less suited for comparison with central carbohydrate metabolism in normal mammalian cells. The latter is more closely reflected by Crabtree-negative, Pasteur-positive yeasts, which favor respiration over fermentation, as a more effective way to generate energy in the presence of oxygen [12,13].

One such yeast, whose genetic investigation is amenable to similar methodologies as those developed for *S. cerevisiae*, is *Kluyveromyces lactis* [14]. In contrast to baker’s yeast, deletions of genes in the upper glycolytic pathway do not impair its growth on glucose, as shown for *KlPGI1* [15] and the two genes encoding the subunits of the hetero-octameric phosphofructokinase, *KlPFK1* and *KlPFK2* [16]. In both cases, an additional deletion of *KlTAL1,* which encodes a key enzyme of the pentose phosphate pathway (PPP), prevented growth on glucose as a sole carbon source [17]. This indicated a strong contribution of the PPP to glucose degradation in *K. lactis*, matching that of glycolysis—a notion substantiated by further genetic and physiological analyses, as recently reviewed [18]. A mutant lacking triosephosphate isomerase due to a deletion in the encoding *KlTPI1* gene also retained the capacity to grow on glucose [19]. A number of glycolytic mutants, including genes encoding a hexokinase and a pyruvate decarboxylase, were obtained in an early screen for *K. lactis* strains resistant to growth in the presence of the respiration inhibitor antimycin A (*rag* mutants; [20,21]). Detailed investigations of the low-affinity glucose transporter Rag1 and its regulation revealed similar components to those involving glucose-sensing in *S. cerevisiae*, albeit with much lower redundancy [22,23]. In the course of these studies, the genes for hexokinase, phosphoglycerate kinase, enolase, and pyruvate decarboxylase were also deleted in *K. lactis* [24]. *Klpgk1* and *Kleno1* deletions were still able to grow on glycerol as a sole carbon source, but failed to grow on glucose or ethanol, as expected. Both deletions impaired the regulation of the *RAG1* gene, identifying the glycolytic flux as an important factor [24]. *RAG1* gene expression is also subject to regulation by oxygen availability, mediated by the transcription factor Sck1 [25]. In contrast, KlHxk1 appears to be more directly involved in mediating glucose repression [26,27]. Homologs of the transcription factors Gcr1 and Gcr2, which strongly contribute to glycolytic gene expression in *S. cerevisiae*, have also been investigated in *K. lactis* [28,29]. A *Klgcr1* mutant was found to mediate resistance to oxidative stress, presumably by redirecting the glycolytic flux into the PPP [28]. Despite its predominantly respiratory metabolism, *K. lactis* disposes of two cytosolic isoforms for alcohol dehydrogenase, whose biochemistry and genetics were elucidated, opening the field for biotechnological applications [30,31,32]. In fact, a lack of the internal mitochondrial NADH dehydrogenase KlNdi1 has been exploited to engineer *K. lactis* for alcoholic fermentation [33]. Despite these fundamental works on central carbohydrate metabolism in *K. lactis*, it remains less well studied by far than in *S. cerevisiae*.

In this context, the reactions of fructose-1,6-bisphosphate aldolase (FBA) and glyceraldehyde-3-phosphate dehydrogenase (GAPDH) were only marginally addressed, although they were of special interest for several reasons. First, these enzymes are located at the key intersections between glycolysis and the PPP. They should thus be required for both pathways and essential for growth on glucose. Neither the *KlFBA1* gene, nor the two homologs encoding NAD^+^-dependent GAPDH isozymes in *K. lactis*, *KlTDH1*, and *KlTDH2* were deleted to test this assumption, until now. Second, an NADP^+^-dependent isozyme of GAPDH, KlGdp1 was discovered in *K. lactis* and the enzyme was characterized after heterologous production and purification [34]. In *K. lactis*, the encoding gene was neither expressed on glucose nor on glycerol, and was proposed to assist in the production of NADPH for growth on pentoses. Again, the *KlGDP1* gene was not deleted in the native host until now. Third, both FBA and GAPDH exert so-called “moonlighting” functions in yeast and other organisms, i.e., biological activities in addition to their catalytic role in glycolysis and gluconeogenesis [35]. For instance, aldolase was shown to interact with the vacuolar ATPase of *S. cerevisiae*, modulating its assembly and activity, in a complex which may also contain GAPDH [36]. Moreover, a physical interaction of the aldolase with RNA polymerase II was suggested to modulate the transcriptional activity towards tRNA genes [37]. In *K. lactis*, FBA has been suggested to participate in the regulation of hypoxic metabolism [38]. A plethora of moonlighting functions have been assigned to FBA in mammalian cells, many related to its interaction with cytoskeleton components (reviewed in [39]). A broad overview of FBA functions apart from its role in central carbohydrate metabolism throughout all biological kingdoms has recently been published [40].

Regarding GAPDH, a number of non-glycolytic functions were discovered for extracellular enzymes in different yeast species. In *S. cerevisiae*, all three isoforms were detected in the cell wall, suggesting an extracellular function [41]. Moreover, the three isoforms were identified as the source of antifungal peptides claimed to ensure the predominance of *S. cerevisiae* in the later stages of wine fermentations [42]. GAPDH from the opportunistic pathogen *Candida albicans* was found in the cell wall as an immunoactive protein, antibodies which can prevent systemic infections caused by this yeast [43]. A homolog of the enzyme was also found in the cell wall of the industrial yeast *Kluyveromyces marxianus* and to mediate flocculation [44]. Other moonlighting functions are suggested by the association of both FBA and GAPDH with mitochondria in high-throughput studies of *S. cerevisiae* [45].

Here, we investigated deletion mutants in *KlFBA1* and the three genes-encoding GAPDH isoforms in *K. lactis*. We showed that KlFba1 is not essential for growth on glucose, but GAPDH activity, mediated by the combined action of KlTdh1 and KlTdh2, is. Promoter fusions with a *lacZ* reporter gene demonstrated that the gene encoding the third GAPDH isoform is repressed by glucose, but expressed on medium containing ethanol as a sole carbon source. The mutants obtained herein provide the basis for heterologous expression of human genes encoding isoforms and disease-related alleles, as exemplified by the human *ALDOA* gene.

## 2. Results

### 2.1. Deletions of KlFBA1 or KlTDH2 Affect Growth on Glucose

A comparison in the yeast gene order browser (http://ygob.ucd.ie/ accessed on 10 December 2021) revealed syntenic homologs of both *FBA1* (encoding fructose-1,6-bisphosphate aldolase) and *TDH2* (encoding a major isoform of glyceraldehyde-3-phosphate dehydrogenase) in the genomes of *Saccharomyces cerevisiae* and *Kluyveromyces lactis*. Consequently, these genes were designated as *KlFBA1* (systematic name KLLA0E07569g) and *KlTDH2* (systematic name KLLA0A11858g). Due to the whole-genome duplication in *S. cerevisiae*, the latter is also syntenic to *ScTDH3*. A further homolog of the dehydrogenase gene annotated in the *K. lactis* genome does not appear to be syntenic with *S. cerevisiae* or the common ancestor, and was designated as *KlTDH1* (systematic name KLLA0F20988g). The deduced protein sequences of ScFba1 and KlFba1 showed 75% identical amino acid residues. KlTdh1 and KlTdh2 were only 80% identical to ScTdh1, but yielded higher values amongst each other and compared to the other two *S. cerevisiae* isoforms (KlTdh1/KlTdh2 = 83%, KlTdh1/ScTdh2 = 82% identity, KlTdh1/ScTdh3 = 81%; KlTdh2/ScTdh2 = 85%, KlTdh2/ScTdh3 = 86%). On the other hand, a gene encoding the previously described NADP^+^-dependent isoform of GAPDH found exclusively in *K. lactis*, *KlGDP1* (systematic name KLLA0F09141g [34]), displayed only approximately 56% identity with the NAD^+^-dependent isoforms of both yeasts.

**Figure 1:**
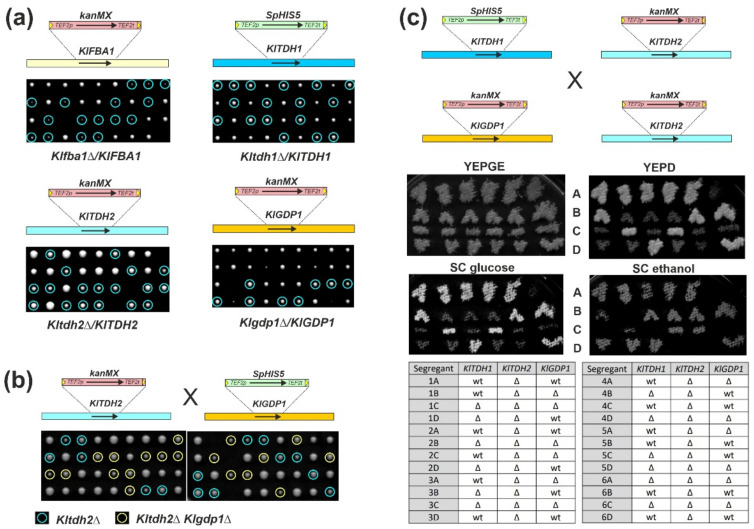
Tetrad analyses of deletion mutants. (**a**) Strains with heterozygous deletions of the genes indicated in the schematic drawings were sporulated and subjected to tetrad analyses on rich medium (YEPD). After micromanipulation, plates were incubated for 2 days (images with smaller colonies) or 3 days (*Kltdh2/KlTDH2*) at 30 °C. Growth was documented by scanning the plates and adjustment of brightness using Corel Photo-Paint. Nine representative tetrads from a total of at least eighteen separated from each diploid are shown. Light-blue circles mark the segregants carrying the respective deletions, as judged from replica plating onto selective marker plates. Note that in the case of the heterozygous diploid for the *Klgdp1* deletion, another deletion, *Kltrp1::kanMX*, carried the same marker cassette. Therefore, only segregants unequivocally carrying the deletion, i.e., those that were both prototrophic for tryptophan and resistant to G418, are highlighted. Strains employed were KHO483 and KHO505 (top row), and KHO455 and KHO461 (bottom row). (**b**) Tetrad analysis of a heterozygous diploid (KHO476) obtained from crossing a *Kltdh2::kanMX* deletion with a *Klgdp1::SpHIS5* deletion. Segregants carrying a single *Kltdh2* deletion are highlighted with a light-blue circle. Double deletions are indicated by yellow circles. The plate was incubated for 3 days at 30 °C after micromanipulation and all 18 tetrads separated are shown. (**c**) Tetrad analysis of a diploid strain heterozygous for both the *Kltdh1* and the *Klgdp1* deletion, but homozygous for the *Kltdh2* deletion (KHO506). After micromanipulation, tetrads were allowed to grow on rich medium with 2% glycerol and 2% ethanol as carbon sources (YEPGE) for 3 days at 30 °C. Segregants were picked onto a master plate of the same medium and allowed to grow overnight. Numbers refer to the tetrad analyzed and signs from top to bottom designate segregants A to D as indicated. Only 6 representative tetrads from a total of 14 tetrads with 4 viable segregants are shown. Growth was documented after replica plating onto the indicated media and incubation over night at 30 °C. The relevant genotype for each segregant can be deduced from the table given below. Segregants carrying *Kltdh1* deletions in combination with *Kltdh2* and/or *Klgdp1* were assigned from their ability to grow on synthetic medium lacking histidine and with 2% glycerol (*w*/*v*) as a sole carbon source. *Klgdp1* deletions were assigned by PCR using flanking oligonucleotides as primers. SC = synthetic complete medium with either 2% glucose (*w*/*v*) or 2% ethanol (*v*/*v*).

To investigate the in vivo function of the encoded enzymes, we proceeded by deleting the four genes from the *K. lactis* genome, as described in Materials and Methods and schematically represented in Figure 1a. Tetrad analyses performed with heterozygous diploids carrying a wild type and a deleted allele of each gene showed that deletions of *KlFBA1* and *KlTDH2* still grow on glucose, albeit yielding smaller colonies than wild-type segregants (Figure 1a). The growth defect was much more pronounced in *Klfba1* deletions than in segregants lacking *KlTDH2*. In contrast, neither deletion of *KlTDH1* nor that of *KlGDP1* affected growth of the respective segregants on glucose (Figure 1a), indicating that the encoded isoforms are dispensable for glucose utilization under standard growth conditions.

In order to determine the importance of the different GAPDH isoforms in vivo, deletions were combined by crossing and the resulting diploids were sporulated and subjected to tetrad analyses. While deletions of *KlGDP1* combined with *Kltdh2* did not produce any additive phenotype with respect to growth on glucose (Figure 1b,c), no viable segregants of the type *Kltdh1 Kltdh2* could be obtained on standard rich medium with 2% glucose (Figure 1c). This suggested that GAPDH activity is required for growth on glucose and that KlGdp1 does not contribute significantly to glucose consumption in vivo. However, *Kltdh1 Kltdh2* double deletions yielded viable segregants on media with non-fermentable carbon sources (2% ethanol plus 2% glycerol; note that glycerol alone suffices as a sole carbon source to promote growth of the double deletions), with similar colony sizes to those of wild-type and single-deletion segregants. Upon replica plating, segregants with the double deletion failed to grow on synthetic media containing 2% glucose but retained the ability to grow on plates with 2% ethanol as a sole carbon source (Figure 1c). In contrast, triple *Kltdh1 Kltdh2 Klgdp1* deletion mutants lacked growth on either glucose or ethanol. Thus, KlTdh1 and KlTdh2 catalyze the reactions from and towards glyceraldehyde-3-phosphate depending on the provided carbon source, while in their absence KlGdp1 activity is sufficient for gluconeogenesis from ethanol.

### 2.2. KlFBA1 Encodes the Sole Fructose-1,6-Bisphosphate Aldolase in K. lactis and KlTdh2 Is the Major Isoform of Glyceraldehyde-3-Phosphate Dehydrogenase

To further substantiate the conclusions drawn from the genetic analyses, specific enzyme activities were determined in crude extracts from different strains obtained in the tetrad analyses. As shown in Table 1, *Klfba1* deletions did not retain detectable FBA activity, while wild-type segregants yielded specific activity of approximately 300 mU/mg protein. This confirms that *KlFBA1* is the only encoding gene and that the enzyme is important, but not essential, for glucose utilization.

On the other hand, *Kltdh2* deletions produced specific GAPDH activities of approximately 25% compared to those of the wild-type strains after growth on rich medium with glucose (Table 1). *Kltdh1* deletions retained more than 50% of wild-type GAPDH activity, confirming that KlTdh2 is the major isoform under standard growth conditions, but KlTdh1 contributes significantly. No GAPDH activity could be detected in *Kltdh1 Kltdh2* double deletions when grown on glycerol or transferred to media with either glucose or ethanol as a sole carbon source (Table 1, and data not shown). Besides lacking any detectable NAD^+^-dependent GAPDH activities, substitution of NADH for NADPH in the reverse enzyme assays did not yield detectable activities in the *Kltdh1 Kltdh2* double deletions or the wild type, even when cells were transferred to ethanol medium 6 h prior to the preparation of crude extracts. This indicates that KlGdp1 activity, although it must be present to allow for growth of the double mutants on ethanol, is extremely low compared to those conferred by KlTdh1 and KlTdh2.

In order to determine if the specific enzyme activities correlate with the expression of the encoding genes, and if the latter depends on the carbon source, we proceeded by fusing the respective gene promoters to the bacterial *lacZ* reporter gene and determined specific ß-galactosidase activities. To this end, the plasmid-encoded reporter constructs were introduced into a recipient *K. lactis* strain lacking its endogenous *Kllac4* gene (Table 2).

Expression from the *KlFBA1* promoter was highest when cells were grown on selective media with glucose as a sole carbon source, with approximately twice the activities driven by the *KlTDH1* and *KlTDH2* promoters, and a very low expression from the promoter of *KlGDP1*. Transfer for six hours to medium with ethanol as a sole carbon source led to an almost eight-fold increase in reporter activity for cells harboring the *KlGDP1p-lacZ* fusion (Table 2). Interestingly, both *KlTDH1-lacZ* and *KlTDH2p-lacZ* constructs also displayed a drastic increase in ß-galactosidase activities on ethanol compared to those on glucose (approximately three- and two-fold, respectively), while those of the *KlFBA1-lacZ* construct slightly decreased (Table 2). This indicates that expression of all genes encoding GAPDH isoforms is subject to different degrees of glucose repression, and that KlTdh1 may play a more important role under gluconeogenic than fermentative growth conditions.

### 2.3. Heterologous Genes Complement the Growth Defects of a Klfba1 Deficiency

**Figure 2:**
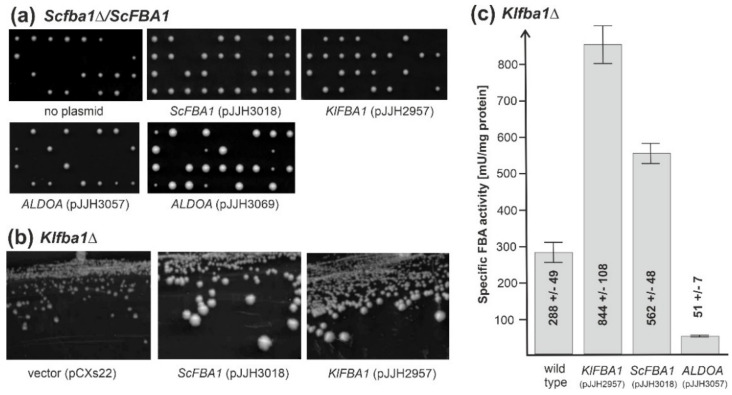
Heterologous production of FBA enzymes in *Saccharomyces cerevisiae* and *Kluyveromyces lactis*. (**a**) A diploid *S. cerevisiae* strain heterozygous for the *Scfba1* deletion (HOD518) was either subjected to tetrad analysis directly (upper panel, left; on YEPGE), or used as a recipient for the plasmids with the genes indicated below each plate (all allowed to germinate on YEPD). Plates were incubated for 2 days at 30 °C, with the exception of the one carrying the *ALDOA* gene on a multicopy vector (pJJH3069), which was incubated for 3 days. Note that growth of more than two segregants per tetrad indicates complementation of the lethal *Scfba1*Δ phenotype. Replica plating onto synthetic media lacking the respective auxotrophic requirements confirmed that segregants carrying the deletion were only able to grow if they carried a plasmid with a complementing gene. (**b**) The haploid *K. lactis* strain KHO477-1D was used to introduce the plasmids indicated and transformants were selected on synthetic medium lacking uracil, with 2% glucose (*w*/*v*) as a carbon source. After preparation of a master plate, cells were streaked out for single colonies on the same medium and incubated at 30 °C for 4 days. Note that the pronounced growth retardation of the recipient strain was complemented by both the native *FBA1* gene and its homolog from *S. cerevisiae*. (**c**) Specific FBA activities were measured in crude extracts from two independent strains, each carrying the plasmids indicated. Cells were grown on synthetic medium with glucose lacking uracil for selection of plasmid maintenance. Values given are the mean of two biological replicas, obtained from at least three technical replicas for each crude extract, with the respective standard deviations (±), which is also represented by the error bars. Strains with a *Klfba1* deletion fail to grow in liquid synthetic medium with glucose and were therefore grown in YEPD, yielding specific activities below detectable levels, as listed in Table 2.

In order to establish yeast as a host for heterologous expression of genes encoding FBA enzymes, we introduced the homolog from *S. cerevisiae* under the control of its native promoter in the *Klfba1* deletion and vice versa *KlFBA1* in the *Scfba1* deletion. Both yeast mutants were also employed to introduce the gene encoding a human FBA isoform (*ALDOA*) under the control of the *ScPFK2* promoter. In tetrad analyses on rich media with glucose, glycerol or ethanol, the *Scfba1* deletion was lethal in our hands. Both the native *ScFBA1* gene and the *K. lactis* homolog restored full growth to the respective segregants in *Scfba1*, when introduced on a *CEN/ARS* plasmid, presumed to reside in 1–2 copies per cell, as judged by colony sizes (Figure 2a). However, *S. cerevisiae* deletions carrying the human *ALDOA* gene did not produce viable progeny when introduced on the low-copy vector but yielded smaller colonies than the wild type when expressed from the *ScPFK2* promoter from the high-copy number plasmid (Figure 2a; lower panel).

Since a *K. lactis* strain lacking its endogenous *KlFBA1* gene can still grow on rich medium with glucose as a carbon source, the two yeast genes under the control of their native promoters were introduced directly into the haploid recipient strain. Transformants were able to complement the growth retardation of the deletion when streaked out for single colonies on synthetic medium with glucose as a carbon source (Figure 2b; note that the same shuttle vectors were employed as for *S. cerevisiae*, but that they are present in higher copy numbers in *K. lactis* due to their pKD1 origin of replication). Accordingly, the genes from both yeast species conferred increased FBA activity to the recipient strain as compared to a wild-type control (Figure 2c). Importantly, the human *ALDOA* gene introduced on the same vector and expressed from the *ScPFK2* promoter also conferred FBA activity, albeit reaching only approximately 18% of that of the wild-type control. This serves as a proof of principle that *K. lactis* is a suitable host for the expression of aldolase genes from various organisms and different modes of catalysis (see Section 3).

### 2.4. KlTdh2, but Not KlFba1, Accumulates in the Nucleus upon Oxidative Stress

**Figure 3:**
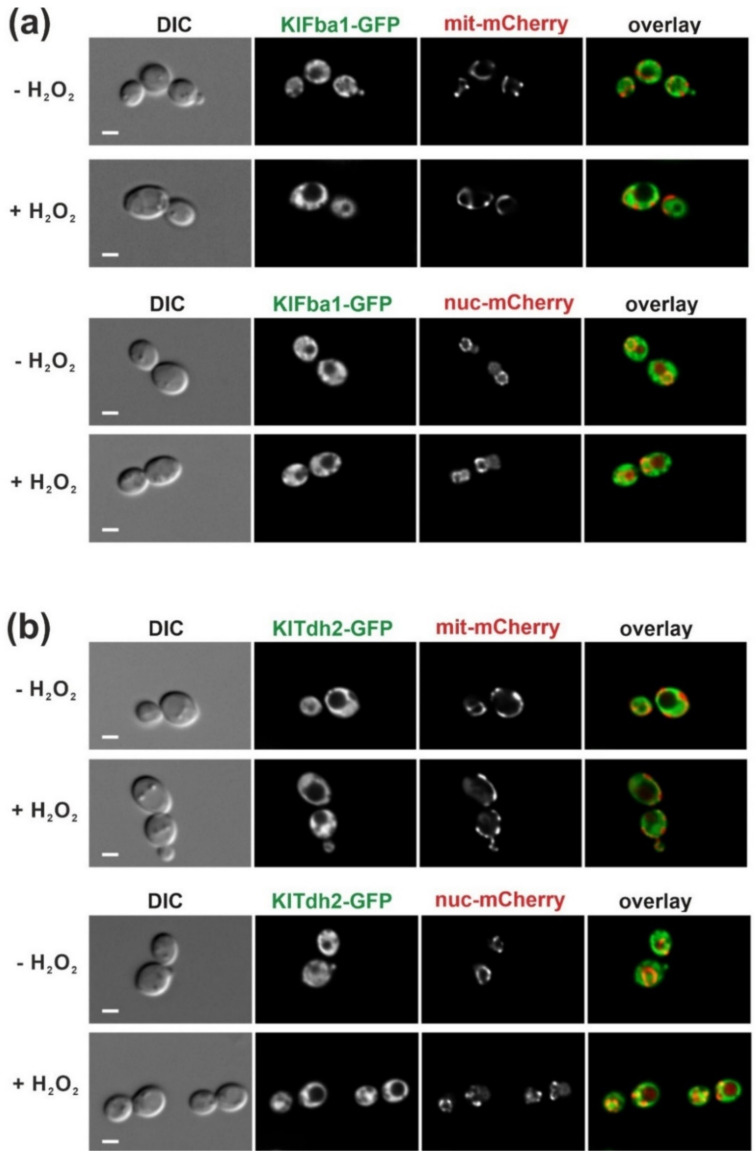
Intracellular distribution of KlFba1 and KlTdh2 with and without oxidative stress. A minimum of 100 cells was examined for each strain and condition and representative images are shown. (**a**) Distribution of KlFba1-GFP in a strain carrying a genomic fusion construct (*KlIDP1-mCherry*) producing a mitochondrial marker (mit-mCherry; upper two panels) or a marker for the nuclear pore complex (*NUP60-mCherry*, nuc-mCherry; lower two panels). Hydrogen peroxide was added at a concentration of 4.4 mM where indicated and images were taken after 20 min of incubation. Distribution of all three marker proteins remained the same after a prolonged incubation for 90 min with hydrogen peroxide. (**b**) Distribution of KlTdh2-GFP in strain backgrounds as in (**a**), i.e., mitochondrial KlIdp1-mCherry (upper-two panels) and nuclear Nup60-mCherry; lower-two panels), without and with 4.4 mM hydrogen peroxide. The latter images were taken after 15 min of incubation with the stressor, in which case the nuclear accumulation of KlTdh2-GFP was still observed in samples taken after 1 h of incubation. Visual inspection of more than 100 cells without and with hydrogen peroxide revealed that nuclear accumulation occurred in more than 75% of the cells exposed to oxidative stress. Strains employed were: KHO474-1A, KHO465-6C, KHO472-1D, KHO466-1A. Size bars correspond to 1 μm for all images in the same panel.

Both FBA and GAPDH enzymes were reported to partially associate with mitochondria under oxidative stress in the *Saccharomyces* Genome Database (SGD) database (https://www.yeastgenome.org/; accessed on 21 December 2021). Therefore, functional C-terminal GFP fusions were constructed for KlFba1 and KlTdh2 encoded at their native loci, and employed in colocalization studies with a mitochondrial marker, KlIdp1-mCherry, in life-cell fluorescence microscopy. As is evident from Figure 3, both enzymes showed a cytosolic distribution under standard growth conditions.

Application of hydrogen peroxide did not provoke a notable accumulation of either enzyme at mitochondria. However, for KlTdh2-GFP a distinct accumulation in a round structure was observed under oxidative stress. Therefore, we employed strains also carrying a genomic *NUP60-mCherry* fusion labeled in a pore protein that resides in the nuclear membrane. The corresponding images confirmed that KlTdh2-GFP accumulated in the nucleus upon oxidative stress (Figure 3b). In contrast, KlFba1-GFP did not display a distinct accumulation in any subcellular compartment.

## 3. Discussion

**Figure 4:**
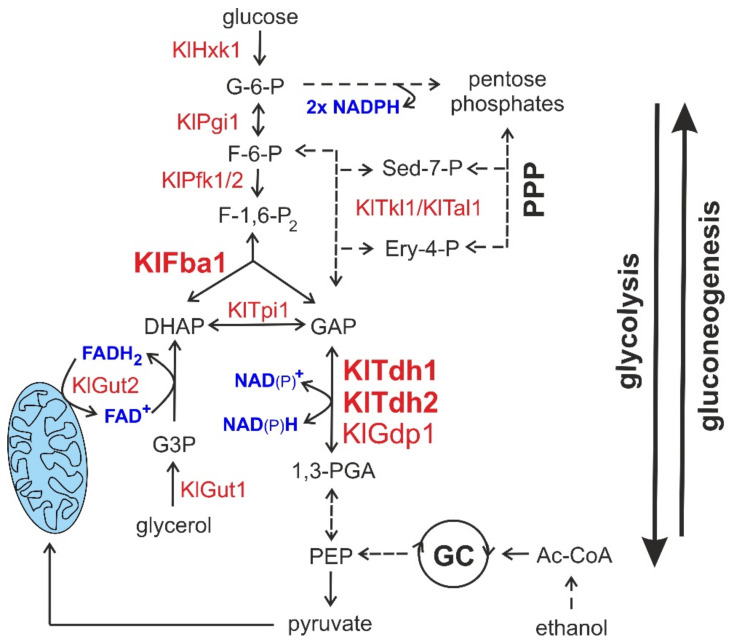
Simplified scheme of carbohydrate metabolism in *K. lactis*. The enzymes discussed in this work are highlighted in large red letters; other important enzymes are designated in smaller red letters. Cofactors are shown in blue. Glycolytic reactions are shown in the center of the upper part and to the right in the lower part. Directions of glycolysis and gluconeogenesis are indicated by bold arrows at the right. PPP designates the pentose phosphate pathway, GC the glyoxylate cycle (note that compartmentalization of the latter reactions is not depicted, here). Mitochondria are represented by the elliptical light-blue structure at the lower left. Dashed arrows indicate the action of multiple enzymes not specified for clarity. For more detailed schemes of the connected pathways, two former works may be consulted [18,47]. Enzyme abbreviations are: KlHxk1 = hexokinase, KlPgi1 = phosphoglucose isomerase, KlPfk1/2 = the hetero-octameric phospho-fructokinase formed by two types of subunits, KlTkl1 = transketolase, KlTal1 = transaldolase, KlFba1 = fructose-1,6-bisphosphate aldolase, KlTpi1 = triosephosphate isomerase, KlTdh1, KlTdh2 = NAD^+^-dependent glyceraldehyde-3-phosphate dehydrogenases, KlGdp1 = NAD^+^/NADP^+^-dependent glyceraldehyde-3-phosphate dehydrogenase, KlGut1 = glycerol kinase, KlGut2 = glycerol-3-phosphate dehydrogenase. Metabolite abbreviations: G-6-P = glucose-6-phosphate, F-6-P = fructose-6-phosphate, F-1,6-P_2_ = fructose-1,6-bisphosphate, DHAP = dihydroxyacetone phosphate, GAP = glyceraldehyde-3-phosphate, G3P = glycerol-3-phosphate, S-7-P = sedoheptulose-7-phosphate, E-4-P = erythrose-4-phosphate, 1,3-PGA = 1,3-bisphosphoglycerate, PEP = phosphoenolpyruvate, Ac-CoA = acetyl-coenzyme A.

Starting from the assumption that glycolysis and the pentose phosphate pathway (PPP) contribute equally to glucose degradation in the milk yeast *Kluyveromyces lactis* [46] (and reviewed in [18]), we decided to investigate the importance of the reactions of fructose-1,6-bisphosphate aldolase (FBA) and glyceraldehyde-3-phosphate dehydro-genase (GAPDH), which are situated at the interface of the two pathways (Figure 4).

In silico analyses suggested that only one gene encoding an FBA was present in the *K. lactis* genome (*KlFBA1*), whereas two homologs encoding putative NAD^+^-dependent GAPDH isozymes were detected (*KlTDH1* and *KlTDH2*), in addition to a gene encoding a previously reported isoform capable of using either NAD^+^ or NADP^+^ (*KlGDP1*; [34]). Deletion of the entire open reading frames of all four genes combined with determinations of specific enzyme activities in crude extracts confirmed that indeed, all isoforms present in *K. lactis* were covered by this study. FBA activity in *Klfba1* deletions was below detectable levels, paralleled by a severe growth defect of such mutants on media with glucose as a sole carbon source. We attribute the slow growth to the fact that the PPP feeds into glycolysis at the levels of fructose-6-phosphate (i.e., prior to the FBA reaction) and glyceraldehyde-3-phosphate (i.e., prior to GAPDH; Figure 4). Some of the carbon from glucose may thus still be directly channeled into the lower part of glycolysis by the PPP in *Klfba1* mutants, while a major part needs to be recycled through the phosphoglucose isomerase reaction and the oxidative part of the PPP.

On the other hand, GAPDH activity is required for sugar degradation by both major pathways, and strains lacking this activity consequently did not grow on glucose. This phenotype also demonstrates that the NAD^+^/NADP^+^-dependent isoform encoded by *KlGDP1* does not contribute under these conditions, most likely because the gene is not expressed, as observed in the reporter fusions. Consistent with previous reports obtained by Northern blots [34], *KlGDP1* transcription is subject to glucose repression. Interestingly, our expression studies revealed that *KlGDP1* is expressed on ethanol medium, albeit only at approximately 10% of the levels observed for the two *KlTDH* gene promoters. This explains why *Kltdh1 Kltdh2* double mutants still grow on ethanol as a sole carbon source, whereas triple *Kltdh1 Kltdh2 Klgdp1* deletions do not. Clearly, KlGdp1 activity alone allows for sufficient gluconeogenesis to support growth in the absence of the two major GAPDH isoforms. It should be noted that we were unable to measure any enzyme activity for KlGdp1 in crude extracts, even when substituting NADH in the reverse enzyme assay for NADPH. We conclude that either the in vitro test conditions were not adequate for the detection of this GAPDH activity, which must be present in vivo, or the enzyme is not sufficiently stable in crude extracts.

Unexpectedly, we observed that the two *KlTDH* genes also appear to be subject to some glucose repression, although to a lower extent than *KlGDP1*. Thus, reporter ß-galactosidase activites from promoter fusions were increased by a factor of 3 (*KlTDH1p*) or two-fold (*KlTDH2p*) when cells were grown on ethanol instead of glucose as a sole carbon source. This indicates an increased requirement for the encoded isoforms under gluconeogenic growth conditions, possibly to allow for sufficient input of metabolites into the PPP, which runs at a higher capacity in *K. lactis* than in *S. cerevisiae* (reviewed in [18]). Replenishment from glyceraldehyde-3-phosphate and fructose-6-phosphate would also avoid excessive production of NADPH by the oxidative part of the PPP, if it was fed only from glucose-6-phosphate generated as the final product of gluconeogenesis while growing on ethanol (Figure 4).

How do *Kltdh1 Kltdh2 Klgdp1* triple mutants generate sufficient energy to grow on glycerol as a sole carbon source? The mitochondrial glycerol-3-phosphate dehydrogenase, KlGut2, which converts glycerol-3-phosphate to dihydroxyacetone phosphate, employs FAD instead of NAD^+^ as a cofactor, generating reducing power directly fed into the respiratory chain (Figure 4). Glucose-6-phosphate and ATP can therefore be generated from glycerol, which represses the glyoxylate cycle and the consumption of ethanol in *K. lactis* [47,48]. The GAPDH isoforms in *S. cerevisiae* were shown to form heteromeric complexes, which may well occur with the *K. lactis* enzymes, too [42]. Concerning the importance of the different isoforms in vivo, KlTdh2 seems to be more reminiscent of ScTdh3, as a lack of the latter also displayed a slight growth defect on glucose media [42]. The authors also proposed another role for GAPDH in yeast sphingolipid metabolism, whose conservation in *K. lactis* remains to be determined.

With regard to the link of carbohydrate metabolism with regulation of gene expression it is rather intriguing that KlTdh2 can accumulate in the nucleus under conditions of oxidative stress. This indicates a possible moonlighting function, which could play a role in transcriptional regulation on top of its function in glycolysis and gluconeogenesis. It will thus be interesting to pursue this hypothesis in future transcriptome and proteome analyses, taking advantage of the deletion mutants generated herein. Another role of GAPDH was observed in the biotechnological application of *S. cerevisiae* for the production of alcoholic beverages [49]. All three GAPDH isoforms were shown to serve as a source for antimicrobial peptides, generated by the metacaspase Mca1 (= Yca1), and were proposed to enhance competition with other fungi and bacteria in wine fermentations. Interestingly, *K. lactis* disposes of a syntenic gene to *ScMCA1* and the two core sequences of the antimicrobial peptides identified in *S. cerevisiae*, SWYDNEYGYS and FRVPTVDVSVVD [49], are conserved in both KlTdh1 and KlTdh2. Whether *K. lactis* shares this antimicrobial capacity and if it could be exploited in its biotechnological applications would be another interesting line of future investigation.

With regard to FBA, the severe growth defect and the lack of detectable enzymatic activity in the *Klfba1* deletion predestine *K. lactis* as a heterologous expression host. The haploid deletion strain, although growing poorly on glucose, can be directly propagated on standard medium and used for the introduction of the homologs from other organisms. Restoration of fast growth can then be directly related to a high complementation capacity, whereas FBA assays in crude extracts can still detect less potent isoforms. This was demonstrated by the expression of *ScFBA1* and the human *ALDOA* gene—which encodes the major isoform in muscle tissue and erythrocytes [50]—in the current study. In this context it is important to note that aldolases come in two fundamentally different varieties: whereas bacteria and fungi use a class II type aldolase which requires a divalent metal (Zn^2+^ in *S. cerevisiae* and presumably also in KlFBA) as a cofactor, insects and vertebrates harbor class I-type enzymes that employ a Schiff-base intermediate in their catalysis [51]. It is thus not surprising that the activity produced by the human clone in the *K. lactis* expression system accounts for less than 20% of the native enzyme with a different mode of catalysis. Nevertheless, it demonstrates that yeasts are a suitable host for expression of genes encoding such fundamentally different enzymes. In *Scfba1* deletions, strong overexpression of the clone was required for complementation. This is consistent with previous reports on the heterologous expression of an *FBA* gene from flies (*Drosophila melanogaster*), where the strong glycolytic gene promoter *PGI1p* and a multicopy plasmid were employed [52]. In the latter work the authors worked with a *Scfba1* deletion able to grow on rich medium with acetate as a carbon source. In the genetic background employed herein, the heterozygous deletion did not produce any viable progeny in tetrad analyses on this or the various other tested media with non-fermentable carbon sources. Although the growth deficiency in *S. cerevisiae* may be overcome by buffering a rich medium with ethanol as a carbon source to pH 5.5 [36], such adaptations are not required for the *Klfba1* strains. We conclude that with the ease of handling and the respiratory metabolism being more similar to that of human cells, *K. lactis* could serve as a better host to study the function of FBA isoforms and alleles associated with human diseases. Since aldolase has been shown to reside in the cell walls of different fungal pathogens, such as *Candida albicans* and related species [53,54,55] and *Cryptococcus neoformans* [56], which contribute to virulence by binding and activating human plasminogen, the enzyme can be a target for drugs and vaccines (reviewed in [40]). With the results presented herein, *K. lactis* could thus be a valuable host with a GRAS (generally regarded as safe) status for high-level production of the respective proteins.

We conclude that both FBA and the different isoforms of GAPDH serve essential functions in the degradation of glucose by the respiratory yeast *K. lactis*. GAPDH activity is further required for gluconeogenesis, for which the isoform encoded by the glucose-repressed *KlGDP1* gene suffices. The nuclear localization of KlTdh2 under oxidative stress provides the first hint to an important moonlighting function of this isoform, which is worth further investigation.

## 4. Materials and Methods

### 4.1. Strains and Growth Conditions

*Kluyveromyces lactis* strains employed and their genotypes are listed in Table 3. All strains derived from the congenic series described in [57], which is based on the type strain CBS2359.

The diploid *Saccharomyces cerevisiae* strain HOD518, with the genotype *MATa/MATalpha ura3-52/ura3-52 leu3-2,112/leu2-3,112 his3-11,15/his3-11,15 fba1::SpHIS5/FBA1*, was constructed in the laboratory strain DHD5 described previously [58] by one-step gene replacement with a PCR-generated *SpHIS5* deletion cassette obtained from pUG27 [59].

Yeast cell culture and genetic techniques followed standard procedures described for *S. cerevisiae* [60]. Rich medium (YEPD) contained 1% yeast extract, 2% Bacto peptone (Difco Laboratories Inc., Detroit, MI, USA), and 2% glucose (all *w*/*v*). Synthetic media were prepared with Difco yeast nitrogen base with ammonium sulfate as described in [60], with the addition of amino acids and bases using a mixture provided by MP Biomedicals (Eschwege, Germany; CSM-His-Leu-Trp-Ura) supplemented as required for selection of plasmids or deletion markers, and 2% glucose (*w*/*v*) as carbon source (SCD). Two percent glycerol (*w*/*v*) and/or 2% ethanol (*v*/*v*) were used as alternative carbon sources in rich or synthetic media as indicated, and 100 mg/L of G418 was added for selection of the *kanMX* marker. Hydrogen peroxide was added at the concentrations indicated to induce oxidative stress.

Crossing of *K. lactis* strains was performed on malt agar plates (5% malt extract, 3% agar, *w*/*v*), incubated for 1–2 days at 30 °C. Diploids were selected by complementation of auxotrophic markers after replica plating onto appropriate synthetic media. They were grown for 8–10 h at 30 °C in 3 mL of YEPD, collected by centrifugation and dropped onto potassium acetate plates (1% potassium acetate, 3% agar, *w*/*v*) for sporulation. After 1–2 days of incubation at 30 °C, tetrad analyses were performed on YEPD plates using zymolyase 20T for digestion of the ascus walls and a Singer Instruments micromanipulator as described in [61]. Strains heterozygous for the *Kltdh1 Kltdh2* double and the *Kltdh1 Kltdh2 Klgdp1* deletions were separated and allowed to germinate on rich medium containing glycerol and ethanol (YEPGE). Plates were incubated for 2–3 days at 30 °C and colony formation was documented by scanning. Brightness and contrast were adjusted for entire plates using Corel Photo Paint.

For manipulations in *E. coli*, strain DH5α was employed with media as described previously [62].

### 4.2. Construction of Plasmids, Gene Fusions and Deletions

For cloning and expression of glycolytic genes in yeast, the triple *K. lactis*/ *S. cerevisiae*/*E. coli* shuttle vector pCXs22 [57] was generally employed. It confers ampicillin resistance to *E. coli* and carries *ScURA3* as a selection marker for both yeasts. A *CEN/ARS* sequence ensures its low-copy number in *S. cerevisiae*, and high-copy numbers in *K. lactis* are conferred by the pKD1 origin of replication. For heterologous gene expression, pJJH2075 was constructed by inserting an optimized *ScPFK2* promoter, described previously [63] as an EcoRI/BamHI fragment, into pCXs22. YEplac181 [64] with a 2 μm origin of replication and an *ScLEU2* marker was used for high-copy number expression of the human *ALDOA* gene in *S. cerevisiae*. For promoter fusions with the bacterial *lacZ* reporter gene, plasmid pJJH2031 was constructed. It carries the entire *lacZ* open reading frame cloned as a PCR-generated BamHI/EcoRI fragment into pCXs18, a low-copy number vector for *K. lactis* from the same series as pCXs22 described above [57,65]. Promoters of genes to be tested were generated by PCR using oligonucleotides with appropriate cloning sites and ligated into the respective sites of pJJH2031 preceding the translational start codon of *lacZ*. All oligonucleotides were custom-made and purchased from Biolegio (Nijmegen, The Netherlands). A concise list of the plasmids derived from these vectors and constructed in this work is given in Table 4.

For assembly and alignments of DNA and protein sequences, the Clone Manager 9 program (Scientific and Educational Software, Denver, USA) was employed. The integrated ClustalW function was used to determine the percentage of sequence identities.

For gene deletions, the entire open reading frames were substituted by selective marker cassettes obtained from pUG6 (*kanMX*) or pUG27 (*SpHIS5*) described in [59]. For this purpose, cassettes were amplified with appropriate oligonucleotide pairs, containing at least 40 nucleotides corresponding to the target sequence at their 5′-end and 20–22 nucleotides of homology to the marker cassettes. PCR products were introduced for homologous recombination into either the diploid recipient strain KHO70 or the haploid strain KHO69-8C, both carrying deletions in the *KU80* gene to reduce the frequency of non-homologous recombination. If this direct strategy of one-step gene deletion failed, the PCR products were employed for in vivo recombination with plasmids carrying the respective genes and longer flanking sequences in *S. cerevisiae*, plasmids were isolated from transformants grown on selective media, amplified in *E. coli*, and fragments with longer homologous regions were introduced into the *K. lactis* strains selecting for the deletion marker. Deletions were verified by PCR with flanking oligonucleotides and obtained in the haploid state for KHO70 by sporulation and tetrad analysis. All strains were routinely back-crossed to *KU80* wild-type strains, sporulated and again subjected to tetrad analyses to avoid genomic instability. All deletions were finally verified by three independent PCRs to ensure their correct integration. Complete sequences of the respective loci are available upon request.

Genomic fusions to genes encoding fluorescence tags followed the same basic strategy as applied for gene deletions, i.e., one-step replacements using PCR products obtained with appropriate oligonucleotide pairs providing homology to the target sequences. Fluorescent marker genes were fused in frame to the 3′-end of the respective coding sequences by eliminating the translational stop codon of the target gene. The fusion cassettes were equipped with a selectable marker, i.e., *kanMX* for GFP (pJJH1619) and *SkHIS3* for mCherry (pJJH1525), with the template vectors described in [66]. Strains with different fluorophores were combined as required for colocalizations by crossing and tetrad analyses.

### 4.3. Fluorescence Microscopy

The setup used for fluorescence microscopy consisted of a Zeiss Axioplan 2 (Carl Zeiss, Jena, Germany) equipped with a 100x alpha-Plan Fluor objective (NA 1.45) and differential-interference contrast. Sample handling and image processing were described in detail in [67]. The setup was controlled by the Metamorph v6.2 program (Universal Imaging Corporation, Downingtown, PA, USA). Brightfield images were acquired as single planes using DIC. Images were scaled using Metamorphs scale image command (Metamorph Offline Version 7.8.0.0, 64-bit). Deconvolution of the fluorescence images was performed by Huygens Remote Manager v3.5. The processed images were overlaid using Metamorphs overlay images command.

### 4.4. Preparation of Crude Extracts and Enzymatic Determination

For preparation of crude extracts, cells were incubated in liquid media overnight with shaking at 30 °C in 5–10 mL of precultures. They were inoculated in 10–20 mL of the media indicated at an OD600 of approximately 1, and incubated for another 6 h. Cells were harvested by centrifugation and washed twice with 2.5 mL ice-cold potassium phosphate buffer (50 mM, pH7.0). Next, 0.5 g of glass beads with a diameter of 0.25–0.5 mm (Carl Roth GmbH, Karlsruhe, Germany) were added together with 0.5 mL of the phosphate buffer and vigorously shaken for 10 min at 4 °C. After addition of another 0.5 mL of ice-cold phosphate buffer, the supernatant was transferred to 1.5 mL Eppendorf tubes kept on ice, and cell debris was pelleted by centrifugation at 13,000× *g* and 4 °C for 10 min. The supernatant was removed into fresh Eppendorf tubes and kept on ice as a crude extract for determination of enzymatic activities. Total protein content was determined by the Micro-Biuret method [68] using bovine serum albumin as a standard. Specific ß-galactosidase activities expressed in nmoles ortho-nitrophenol-ß-D-glucopyranoside (ONPG) hydrolyzed per min and mg protein at 30 °C (mU) were determined, as described previously [69]. FBA and GAPDH activities were determined by coupling the conversion of substrates to the oxidation of NADH by ancillary reactions, with the addition of salts and cofactors as listed in [70]. Briefly, fructose-6-phosphate was used at 2 mM as a substrate for FBA, and the fructose-1,6-bisphosphate produced was converted by the ancillary enzymes triosephosphate isomerase and glycerol-3-phosphate dehydrogenase, consuming NADH. For GAPDH assays, the reverse reaction to glycolysis was employed. Thus, 3-phosphoglycerate was used at 10 mM concentration in conjunction with 1 mM ATP to be converted by the ancillary enzyme phosphoglycerate kinase to 1,3-bisphosphoglycerate, which served as a substrate for the GAPDH reaction. Note that in the current assays, 5 mM cysteine was replaced by 20 mM dithiothreitol as a reducing agent. All specific activities are expressed as nmoles of substrate converted per min and mg of protein at 30 °C (mU/mg). To test for KlGdp1 activity, NADH was replaced by NADPH. All enzyme assays were carried out in a Beckman DU800 photometer (Beckman-Coulter, Krefeld, Germany) with an automated cell changer kept at 30 °C, recording the decrease in adsorption at 340 nm for 10–15 min.

## Figures and Tables

**Table 1:** Specific activities of fructose-1,6-bisphosphate aldolase (FBA) and glyceraldehyde-3-phosphate dehydrogenase (GAPDH) in *K. lactis* wild-type and deletion strains.

Enzyme	Strain	Relevant Genotype	Specific Activity ^1^ [mU/mg Protein]
**FBA**	KHO46-2A	wild type	303 ± 6
	KHO285-1A	wild type	268 ± 35
	KHO452-6C	*Klfba1*Δ	<1
	KHO489-6B	*Klfba1*Δ	<1
			
**GAPDH**	KHO502.2-3D	wild type	1069 ± 133
	KHO502.2-5B	wild type	1080 ± 157
	KHO502.2-17C	*Kltdh1*Δ	597 ± 65
	KHO502.2-17D	*Kltdh1*Δ	547 ± 36
	KHO502.2-1A	*Kltdh2*Δ	238 ± 23
	KHO502.2-17A	*Kltdh2*Δ	256 ± 18
	KHO502.2-2C	*Kltdh1*Δ *Kltdh2*Δ	<2
	KHO502.2-5D	*Kltdh1*Δ *Kltdh2*Δ	<2

^1^ Specific activities were routinely measured in crude extracts from strains grown in rich medium with 2% glucose (*w*/*v*) as a carbon source (YEPD). In the case of *Kltdh1 Kltdh2* double deletions, which cannot grow on glucose, the hexose in the growth medium was substituted by 2% ethanol (*v*/*v*) as a carbon source. GAPDH activities were determined in the reverse direction to glycolysis as described in materials and methods. Activities given represent the mean values of at least three independent determinations from each crude extract with the standard deviations (±).

**Table 2:** Specific ß-galactosidase activities obtained from the expression of *lacZ* reporter constructs.

Plasmid	Promoter	Specific Activity [mU/mg Protein] ^1^	
		2% Glucose	2% Ethanol
pJJH3043	*KlFBA1p*	224 ± 6	162 ± 4
pJJH3061	*KlTDH1p*	118 ± 8	363 ± 13
pJJH3042	*KlTDH2p*	127 ± 6	222 ± 8
pJJH3041	*KlGDP1p*	3 ± 1	22 ± 1
pJJH2031	none	<1	<1

^1^ Strains were grown after inoculation from a preculture for 6 h in logarithmic phase on selective synthetic medium lacking uracil with the indicated carbon sources. Specific ß-galactosidase activities were measured twice in crude extracts from two independent transformants, each. Standard deviations are given (±). KHO209-8A was used as a recipient strain for all constructs. It lacks intrinsic ß-galactosidase activity due to a deletion of the *KlLAC4* gene.

**Table 3:** *K. lactis* strains used in this work.

Strain ^1^	Genotype
**Haploid:**	
KHO46-2A	*MATalpha ura3 his3::loxP*
KHO69-8C	*MATalpha ura3 leu2 his3::loxP ku80::loxP*
KHO209-8A	*MATa ura3 leu2 his3::loxP lac4::loxP*
KHO285-1A	*MATa his3::loxP*
KHO452-6C	*MATalpha ura3 leu2 fba1::kanMX*
KHO465-6C	*MATa ura3 leu2 ade4::loxP KlFBA1::GFP-kanMX KlNUP60::mCherry-kanMX*
KHO466-1A	*MATalpha ura3 leu2 his3::loxP KlTDH2::GFP-kanMX KlNUP60::mCherry-kanMX*
KHO472-1D	*MATa leu2 his3::loxP ade4::loxP KlTDH2::GFP-kanMX KlIDP1::mCherry-SkHIS3*
KHO474-1A	*MATa ura3 leu2 his3::loxP KlFBA1::GFP-kanMX KlIDP1::mCherry-SkHIS3*
KHO477-1D	*MATa ura3 fba1::kanMX*
KHO489-6B	*MATa leu2 fba1::kanMX*
KHO502.2-1A	*MATalpha leu2 his3::loxP ade4::loxP Kltdh2::kanMX*
KHO502.2-2C	*MATalpha leu2 his3::loxP ade4::loxP Kltdh1::SpHIS5 Kltdh2::kanMX*
KHO502.2-3D	*MATa ura3 leu2 his3::loxP ade4::loxP*
KHO502.2-5B	*MATalpha ura3 leu2 his3::loxP ade4::loxP ku80::loxP*
KHO502.2-5D	*MATalpha leu2 his3::loxP Kltdh1::SpHIS5 Kltdh2::kanMX*
KHO502.2-17A	*MATa leu2 his3::loxP ade4::loxP Kltdh2::kanMX*
KHO502.2-17C	*MATalpha ura3 leu2 his3::loxP Kltdh1::SpHIS5*
KHO502.2-17D	*MATalpha ura3 leu2 his3::loxP Kltdh1::SpHIS5*
	
**Diploid:**	
KHO70	*MATa/MATalpha ura3/ura3 leu2/leu2 his3::loxP/HIS3 ade2::loxP/ADE2 ku80::loxP/ku80::loxP*
KHO455	*MATa/MATalpha ura3/URA3 leu2/LEU2 his3::loxP/HIS3 ade4::loxP/ADE4 Kltdh2::kanMX/KlTDH2 ku80::loxP/KU80*
KHO461	*MATa/MATalpha ura3/URA3 leu2/LEU2 his3::loxP/HIS3 trp1::kanMX/TRP1 Klgdp1::kanMX/KlGDP1 ku80::loxP/KU80*
KHO476	*MATa/MATalpha leu2/LEU2 his3::loxP/his3::loxP Kltdh2::kanMX/KlTDH2 Klgdp1::SpHIS5/KlGDP1*
KHO483	*MATa/MATalpha ura3/URA3 leu2/LEU2* *Klfba1::kanMX/KlFBA1*
KHO505	*MATa/MATalpha ura3/URA3 leu2/LEU2 his3::loxP/HIS3 Kltdh1::SpHIS5/KlTDH1 ku80::loxP/KU80*
KHO506	*MATa/MATalpha leu2/LEU2 his3::loxP/HIS3 Kltdh1::SpHIS5/ KlTDH1 Kltdh2::kanMX/KlTDH2 Klgdp1::kanMX/KlGDP1*

^1^ Strains KHO46-2A, KHO69-8C, and KHO70 have been described previously [57]. All other strains were constructed in this work.

**Table 4:** Plasmids constructed in this work.

Plasmid	Vector	Inserted Sequences ^1^
***FBA* gene expression**		
pJJH2957	pCXs22	*KlFBA1* with its flanking sequences cloned as an EcoRI/BamHI fragment
pJJH3018	pCXs22	*ScFBA1* with its flanking sequences cloned as an EcoRI/BamHI fragment
pJJH3057	pJJH2075	Synthetic-yeast-optimized human *ALDOA* cloned as a BamHI/HindIII fragment under control of *ScPFK2p*
pJJH3069	YEplac181	*ScPFK2p-ALDOA* subcloned from pJJH3057 as an EcoRI/BamHI fragment
		
***lacZ* fusions**		
pJJH3041	pJJH2031	*KlGDP1* promoter cloned as a HindIII/BamHI fragment
pJJH3042	pJJH2031	*KlTDH2* promoter cloned as a HindIII/BamHI fragment
pJJH3043	pJJH2031	*KlFBA1* promoter cloned as a HindIII/BamHI fragment
pJJH3061	pJJH2031	*KlTDH1* promoter cloned as a HindIII/BamHI fragment
		

^1^ All DNA fragments to be inserted were generated by PCR from genomic yeast DNA using oligonucleotides with the appropriate restriction sites. As an exception, the human *ALDOA* coding sequence was obtained by custom synthesis from GeneArt (ThermoFisher Scientific, Germany) with codons optimized for *S. cerevisiae*. All sequences were confirmed after cloning by custom Sanger sequencing (Seqlab, Göttingen, Germany) and the entire plasmid sequences and detailed descriptions of their constructions are available upon request.
